# Sensory and Affective Dimensions of Pain and Anxiety Like Behaviors Are Altered in an Animal Model of Pain Empathy

**Published:** 2019-07

**Authors:** Masoud Nazeri, Goli Chamani, Fatemeh Abareghi, Fatemeh Mohammadi, Mohammad-Hosseyn Talebizadeh, Mohammad-Reza Zarei, Mohammad Shabani

**Affiliations:** 1 Department of Oral Medicine, Orofacial Pain and Headache Clinic, School of Dentistry, Kerman University of Medical Sciences, Kerman, Iran.; 2 Oral and Dental Diseases Research Center, Kerman University of Medical Sciences, Kerman, Iran.; 3 Department of Neuroscience, Kerman Neuroscience Research Center, Neuropharmacology Institute, Kerman University of Medical Sciences, Kerman, Iran.

**Keywords:** *Anxiety*, *Conditioned Place Aversion (CPA)*, *Empathy*, *Pain*

## Abstract

**Objective:** Pain is a unique and subjective experience that has a prominent function in animals’ survival. Observation of pain in others leads to alterations in pain sensation and affection, termed “Empathy for pain”. The present study aimed to evaluate the effect of empathy on sensory and affective dimensions of pain and its effect on anxiety-like behaviors.

**Method** **:** In this study, male Wistar rats were used. Two cage mates were selected, one of which underwent administration of a noxious stimuli for 10 days and the other observed the conspecific in pain. Hot plate, tail flick, and conditioned place aversion were used to evaluate sensory and affective dimensions of pain, respectively. Anxiety-like behavior was assayed using elevated plus maze paradigm and time spent in open and close arms and number of entrance into each arm was recorded as the anxiety indicator within a 5-minute framework.

**Results: **Rats observing the cage mate in pain had a lower threshold to noxious stimuli in comparison to controls. They also had an increased aversion from painful stimuli, demonstrating heightened affective response to pain. Anxiety-like behavior was also enhanced in the observers.

**Conclusion: **Results of this study demonstrate that both sensory and affective dimensions of pain are altered following observation of pain in a conspecific. Further studies evaluating the underlying mechanisms are encouraged to elucidate the role of different neurotransmitters in this phenomenon.

Empathy is defined as the mental capability to comprehend and respond to the feelings and emotions of other people accordingly. Although empathy has long been considered as a human trait and depends on theory of mind in the mankind, recent studies have revealed a longer neuro-evolutionary lineage for this cognitive ability. Empathy for pain means that following an observation of pain in a conspecific, similar sensations and feelings are experienced by the observer.

This is a special kind of empathy, which is thought to exist throughout a wide range of animals other than humans (1-6). 

Pain is a multidimensional phenomenon possessing both sensory-discriminative and affective motivational dimensions. The sensory dimension of pain is correlated with the intensity of the noxious stimulus and acts to protect the organism, while the affective-motivational dimension recruits higher order brain regions and serves to value the threat of noxious stimuli and the cognitive volitional response to the stimulus. 

It is demonstrated that empathy for pain activates brain regions responsible for affective processing of pain in humans, but no study has studied this dimension of pain in the animal models for empathy (7). Even further, in subjects with congenital insensitivity to pain, empathy for pain directly activates brain regions corresponding to affective dimension of pain, revealing distinct underlying mechanisms for sensory and affective dimensions of pain in both first-hand experience of pain and empathy for pain (8, 9). Many studies have previously focused on sensory dimension of pain, but neither has addressed the affective pain alterations following empathy in animals (10).

Langford et al were the first to describe empathy-like responses in rats (11), and further studies demonstrated that observation of pain in a conspecific leads to enhanced response to noxious stimuli of different modality (11-14). Despite this, the affective aspect of pain is not well-addressed in previous studies and the need for animal models to study the affective motivational dimension of pain in empathy is felt immensely.

According to previous studies regarding the effect of empathy for pain on sensory dimension of pain in rodents and lack of evidence regarding the effect of empathy on affective features of pain in the observer, this study was performed to evaluate the effect of observing a cage mate in pain on cognition, affective pain, and anxiety-like behavior of the observer animal. 

## Materials and Methods

Adult male Wistar rats (weighing 250-270 g) were used in this study. Animals were held in groups of 2 in standard cages one month prior to the initiation of experiment. Animals were given ad libitum access to food and water and were kept in standard environmental conditions (25˚C room temperature, 12/12 light dark cycle) in the animal house of Kerman Neuroscience Research Center. All the efforts were made to minimize harm to the animals and all the procedures were approved by the Ethics Committee of Kerman University of Medical Sciences (Ethics code: KNRC/ 96-5) (15).

Animals were kept together in shared cages 1 month prior to the initiation of experiments to get accustomed to each other. One of the animals was randomly assigned to formalin (pain) or saline injection (control) and the other animal in the same cage was chosen as the observer. Another set of animals with no manipulations were chosen as the naïve group. 

In the first day of the experiments, animals were brought to the laboratory environment. Pain or control animals received an injection of subcutaneous formalin (50µL, 25) in the hind paw (16) or saline (50µL), respectively. Immediately after injection, animals were placed in their cages, adjacent to their cage mates for direct observation of the injected animals’ behavior. This procedure was continued for the next 10 days. 

On the 11th day, animals were again brought to the testing lab and the following tests were performed on them. There was a 2-hour interval between each paradigm. 

***Elevated Plus Maze (EPM)***

This paradigm assesses the anxiety-like behavior of the animals. EPM apparatus was made of wood with 2 open and 2 closed arms and height of 50cm. Animals were placed in the center of the arms and their behavior was recorded using a camera installed above the EPM apparatus. The following parameters were recorded for each animal: number of entrances into open and closed arms, and the time spent in open and closed arms (15). 

***Hot Plate and Tail Flick***

In this procedure, animals’ responses to noxious heat stimuli were evaluated. The hot plate apparatus (LE710 Model, LSI LETICA and Spain) was set at 52 ± 0.5 ˚C. The device has a Plexiglas wall 30cm high and is made of a metal plate 19cm in diameter. The period since the placement of animals into the device until the observation of pain responses (hind-paw licking or jumping) was considered as the reaction time (RT) for each animal. A cut-off of 45s was used to avoid tissue damage (17). 

Tail flick that assays the response to acute thermal stimuli was applied to the tail region. It is a reflex-protective response demonstrating the responsiveness of neurons at the spinal level. Animals were restrained in a plastic restrainer and were allowed to habituate for 30 minutes. Their tails were free and hanging outside the restrainer. The lower 5cm portion of the tail was marked and placed under an intense light, and the period since light eminence until the observation of tail movement was recorded for each animal (17).

***Conditioned Place Aversion (CPA)***

This paradigm was used by Johansen et al (2001) to evaluate the affective dimension of pain, and many other studies have used the same procedure so far (18). The CPA apparatus consists of 3 parts (45 × 45cm each) made of Plexiglas. Two of the 3 compartments were painted with black lines (vertical or horizontal) and the middle portion had white walls (Neutral portion). In the preconditioning day, animals were placed in the neutral portion and the guillotine doors were opened. Animal entrance into each compartment was recorded, and if there was not a significant bias to 1 compartment (time spent in 1 compartment more than 60% of the total time), animals were enrolled in the CPA paradigm. Preconditioning was 20 minutes for each rat. On the conditioning days (days 2-5), animals were injected with subcutaneous formalin (50µL, 2%) into the hind paw in 1 compartment or saline (50µL) in the other room so that each animal received formalin for 2 days matched with 1 compartment and was allowed to explore the compartment for 50 minutes.

On the post-conditioning day (day 6), animals were again placed in the neutral portion and the doors were opened. The time spent in each compartment was recorded during a 20-minute interval. The CPA score was calculated using the following formula:

CPA Score = (Time spent in the formalin-matched compartment in the post-conditioning day) – (the time spent in the formalin-matched compartment in the preconditioning day).

The CPA score was separately calculated for each animal as an indicator of aversion to the reminders of noxious stimuli, demonstrating the magnitude of affective dimension of pain. 

***Statistical Analysis***

Data were analyzed using SPSS V.16 (IBM, Texas and USA). ANOVA with Tukey’s post-hoc test were used to compare data between different groups. P<0.05 was considered statistically significant.

## Results

**Figure 1:**
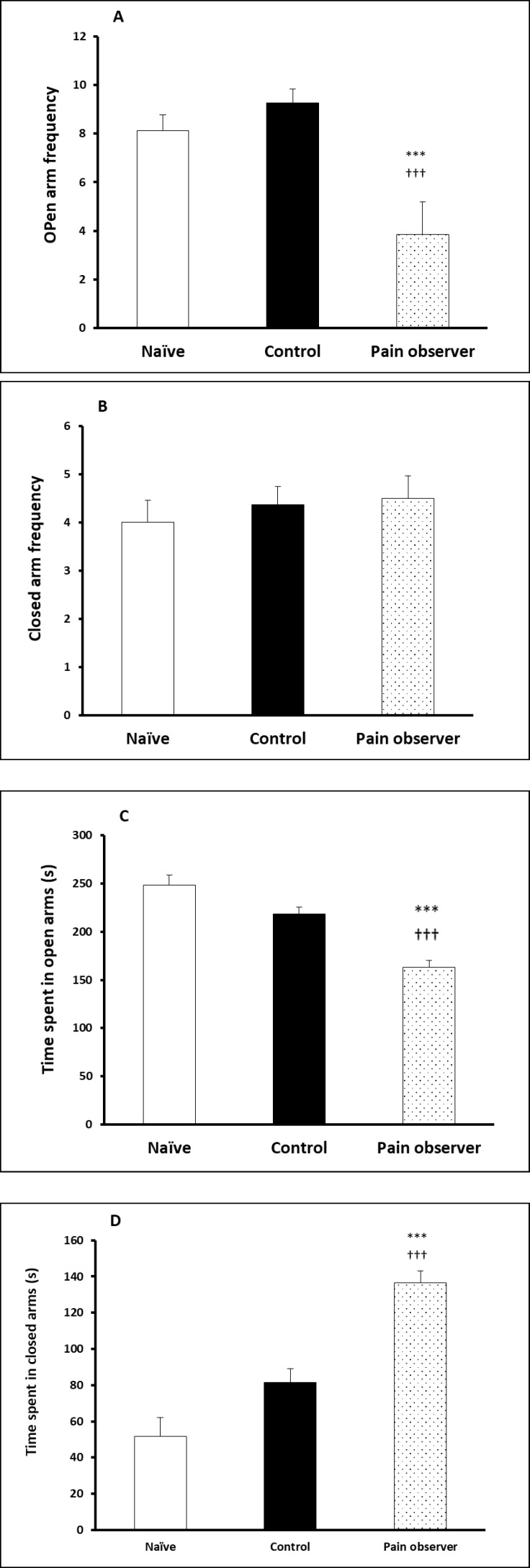
(A and B) Number of Entrances into the Open Arm Was Decreased in Pain Observing Group, Indicating a Higher Level of Anxiety. (C and D) Time Spent in Open and Close Arms Was Significantly Decreased and Increased, Respectively, in Empathy Group, Demonstrating an Increased Anxiety-Like Behavior in the Animals. *** p<0.001 and †††p<0.001, ANOVA Followed by Tukey’s Post-Hoc.

Animals observing a cage mate in pain had an increased anxiety-like behavior in the EPM paradigm. The number of entrances into open arms was significantly reduced in the pain-observing group in comparison to the controls and naïve animals. Furthermore, the number of entrances into the open arms was significantly increased in the pain-observing group in comparison to the other 2 groups (Figure1.A and B) (p = 0.0009, ANOVA).

The time spent in open arms and close arms was significantly different in the pain-observing group in comparison to the other 2 groups (Figure1.C and D) (p = 0.0004, ANOVA). These findings demonstrate an increased anxiety-like behavior in the animals following observation of pain in a cage mate. 

**Figure 2:**
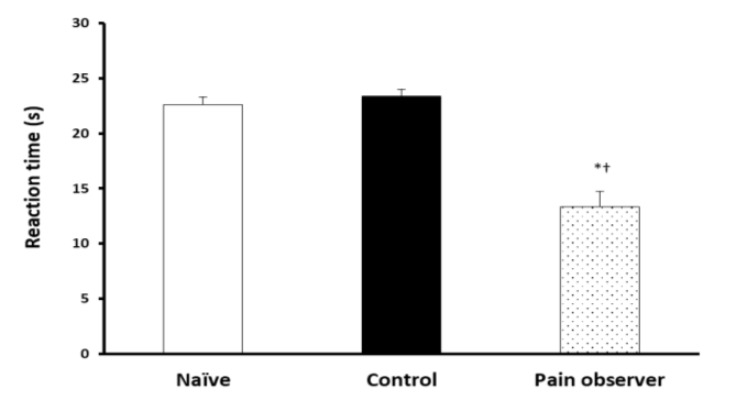
A Decreased Thermal Nociception Threshold Was Observed in the Empathy Group in Comparison to other 2 Groups, Implying a Hyperalgesic Effect for Empathy * p<0.05 and †††p<0.001, ANOVA Followed by Tukey’s Post-Hoc.

**Figure 3:**
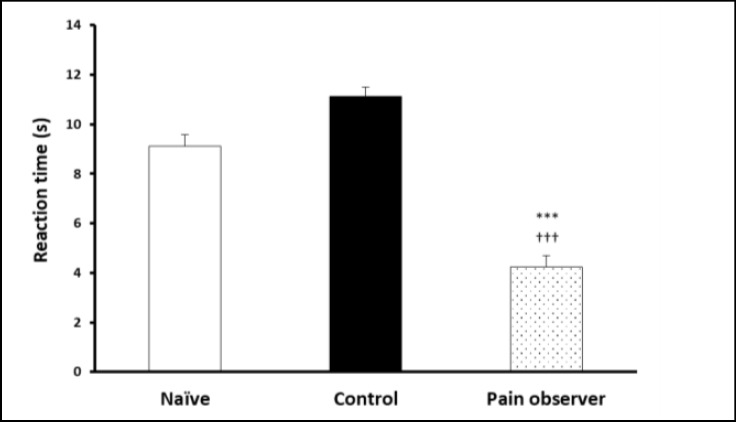
Decreased Acute Thermal Nociception Threshold in Tail Flick Revealed That Spinal Nociception Is Enhanced Following Empathy *** p<0.001 and †††p<0.001 in Comparison to Naïve and Control Animals.

Reaction time for both hot plate (Figure 2, p = 0.018; ANOVA)) and tail flick (Figure 3, p = 0.001; ANOVA) assays were significantly reduced in the pain-observing group in comparison to control and naïve animals.

**Figure 4:**
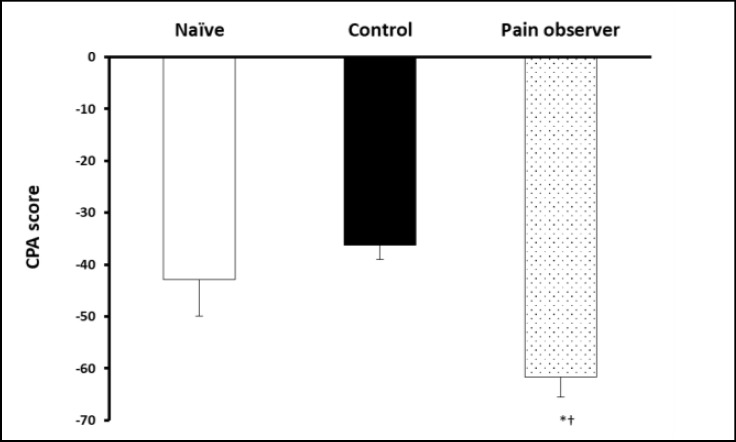
Conditioned Place Aversion (CPA) Score of Empathy Group Was Significantly Increased in Comparison to the other 2 Groups, Revealing an Increased Affective Response. * p<0.05 and †p<0.05, ANOVA, Followed by Tukey’s Post-Hoc.

The CPA score was significantly different from control and naïve groups (Figure 4, p = 0.03; ANOVA), demonstrating that animals observing a cage mate in pain have an increased affective pain response in comparison to other 2 groups. 

## Discussion

Results of this study demonstrated that following direct observation of pain in a cage mate, rats’ nociception and anxiety-like behavior changed dramatically. Furthermore, affective dimension of pain, as revealed by CPA paradigm, was altered following observation of pain in a conspecific, which is the most important finding of the current study.

Empathy for pain has been shown to exist in animals other than humans. Chimpanzees, monkeys, and even rats demonstrate such empathy for pain (19, 20). Interestingly, following observation of a noxious stimuli applied to a conspecific, response to other modalities of nociceptive stimuli (such as thermal or electrical) changes, which demonstrates that central mechanisms are responsible for these alterations (11). Mogil et al were the first to report alterations in pain response following the application of noxious stimuli to a cage mate. They found that the first animals taken from a cage had a higher threshold for pain in comparison to the animals taken from the same cage after the return of the first animal to the cage (13, 14). This finding led to the discovery of empathy-like behaviors in rats by Langford et al (2006), who demonstrated that pain response of different modalities is altered following observation of pain in a conspecific (13, 14). The reason for conducting experiments on the cage mates came from the observations of Mogil et al and Lu et al. (2017), who demonstrated that being a cage mate is important in invocation of empathy in the animals (11-13, 21).

Empathy might invoke several emotional responses in animals. In this study, anxiety-like behaviors were significantly increased in the pain observing animals. This shows that empathy for pain alters emotional processes as well. One of the mechanisms involved might be the changes in different brain regions. Singer et al (2004) demonstrated that brain regions responsible for affective dimensions of pain demonstrate altered activation patterns following empathy in humans (7, 22). One of the most important of these regions is anterior cingulate cortex (ACC), which is also involved in emotional response to noxious stimuli and modulation of affect and mood, including hedonic evaluation and behavioral prediction. Furthermore, it has been demonstrated that ACC function is altered in anxious subjects (23). Thus, it might be plausible to observe that empathy alters anxiety-like behaviors. Furthermore, more studies should be conducted to investigate the role of different brain regions in anxiety-like behavior alterations following empathy for pain and also different neural circuitry for these observations should be elucidated in future studies.

Increased response to noxious stimuli following empathy has been reported in previous studies, which is consistent with the findings of the current study (11, 24). This increased response might be due to modulation of descending modulating systems or central changes leading to exaggerated response to noxious stimuli. Pain modulating system consists of the higher order CNS regions which lead to the modulation of noxious stimuli at the spinal level’s dorsal horn; and one of the most important modulators of this pathway is the Opioidergic system. One of the most recent findings in the field of empathy has demonstrated that following the administration of acetaminophen as a central acting analgesic, empathy for pain decreases, which might be due to the activation of this modulating system at the higher order level (e.g., ACC) (25). This clearly demonstrates that central mechanisms are activated in empathy-induced hyperalgesia. Further studies are needed to evaluate the role of different neurotransmitters in this phenomenon.

The most important finding of the current study was that the affective component of pain is increased following the observation of pain in a cage mate. Johansen et al have demonstrated that ACC lesions lead to impaired affective pain response (18). The role of ACC in emotional regulation, executive function, and pain perception has already been demonstrated (26). Furthermore, Singer et al (2004) have demonstrated that ACC is one of the most distinct brain regions activated following observation of pain in another human (7). Now the question is whether or not ACC functional changes are directly involved in affective pain alterations following empathy.

## Limitation

This study had some limitations. Evaluating the role of different neurotransmitters and the neurobiological basis of empathy-induced changes should be conducted in further studies. Using CPA to evaluate affective dimension of pain has been done in previous studies, but the fact that affective dimension of pain in humans is much more complicated leads to the conclusion that these kinds of studies should also be conducted on human subjects as well.

## Conclusion

This study demonstrated that affective dimension of pain is altered following the observation of pain in a conspecific, which implies that in addition to sensory changes following empathy for pain, more elaborate cognitive functions are also affected by empathy. These findings provide a strong background for evaluating the role of brain regions corresponding to affective dimensions of pain, including prefrontal cortex, and ACC.
